# Patients experiences of maintaining mental well-being and hope within motor neuron disease: a thematic synthesis

**DOI:** 10.3389/fpsyg.2015.00606

**Published:** 2015-05-12

**Authors:** Andrew Soundy, Nicola Condon

**Affiliations:** ^1^Department of Physiotherapy, School of Sport, Exercise and Rehabilitation Sciences, University of BirminghamBirmingham, UK; ^2^University Hospitals Birmingham, NHS Foundation TrustBirmingham, UK

**Keywords:** qualitative, review, motor neuron disease, experiences, hope, adjustment

## Abstract

Research is required that can synthesize the experiences of patients with Motor Neuron Disease (MND). One value of being able to do this is to understand the psychological experiences and processes involved in maintaining mental well-being and hope. A qualitative thematic synthesis of studies was undertaken. Studies were electronically searched from inception until June 2014. Twenty-nine studies with 342 (175 male) unique individuals with MND were identified. Five themes were identified: (1)The effects of the disease on interactions, relationships, roles and meaningful activities, (2) Responses that relate to the expression of hope, (3) Factors which disable hope, (4) Factors which enable hope, and (5) Cognitive and Practical adaptation that enabled hope, control and coping. Finally, a model of hope enablement was identified that considers the psychological pathways undertaken by a patient which influence mental well-being and hope. Within this review article evidence is provided which illustrates the central importance of relationships and social support for individuals with MND. Further, it has been identified that periods of coping are possible and are likely associated with greater mental well-being for patients with MND.

## Introduction

Motor neuron disease (MND) is a progressive illness which involves the degeneration of lower and upper motor neurons (McLeod and Clarke, 2007). Amyotrophic Lateral Sclerosis (ALS) is the most common form of MND, presenting in over 75% of cases (Lillo and Hodges, 2009), but, for the purposes of this article will be used synonymously with it. Peak onset is between 47–63 years for the familial disease and 47–52 years for the sporadic disease and often has an unpredictable progression thereafter (Kirenan et al., 2011). A general prevalence has been identified in Europe as approximately 2/100,000 (Logroscino et al., 2009) and in the US as 4/100,000 (Mehta et al., 2014). Patients with MND face physical and psychological challenges, including high symptom burden (for instance fatigue, dyspnoea, and pain) as well as the loss of independence and the inability to communicate (Blackhall, 2012).

The psychological aspects of the disease have traditionally been less researched and calls for future research have been suggested to focus on improving and maintaining a patients quality of life (McLeod and Clarke, 2007), of which mental well-being has an integral role. Mental well-being is defined as a sense of satisfaction, optimism and purpose in life, a sense of mastery, control, belonging, as well as the perception of social support (National Health Service Scotland, 2014). A lack of meaning in life, a central element of mental well-being, has been identified as a predictor of hopelessness for patients with MND (Plahuta et al., 2002; Blackhall, 2012). Hopelessness is one psychological aspect which presents a significant problem for patients with MND (Averill et al., 2007) and is a strong predictor of suicidal ideation (McLeod and Clarke, 2007). The ability to (re)access central aspects of mental well-being may be explained by the recent development of a model of hope enablement (Soundy et al., 2014d), which revolves around the importance of re-establishing an individual's generalized hopes.

Generalized hopes include hopes which exist on different levels, these levels include hope in the following aspects of life; hope for a relief in suffering, hopes which relate to an individual's social roles and responsibilities, hopes which are generated by meaningful and more superficial activities, tasks, accomplishments and interactions (Soundy et al., 2014a,d). It's very likely that the losses experienced by patients with MND exist across the different levels of generalized hopes. For instance, major losses include social interaction, isolation and role changes (McLeod and Clarke, 2007), as well as challenges to independent living (Ozanne et al., 2011). However, positive coping experiences are possible and can be framed around relationships, occupation, employment, leisure activities (Young and McNicoll, 1998). Considering how to initiate, change, improve or restore such losses is essential to maintain a patient's mental well-being. The current model of hope enablement (Soundy et al., 2014a) falls short of considering the processes of adjustment which follow the experience of loss. In particular no consideration is made to the expression of hope, the ability to access adaptive responses or consideration regarding the cognitive processes that an individual may go through following the experience of loss.

The expression of hope (Soundy et al., 2013) in individuals with chronic neurological conditions is directly associated with the adoption of hope or coping strategies which enable access to generalized hopes. Three expressions of hope has been identified in patients with neurological conditions; (1) as a paradox (an expression which illustrates that patients are able to simultaneously accept and defy aspects about their illness and situation), (2) as a dichotomy (considered as either a concrete hope for a cure or complete restoration, or as no hope for improvement, informed by a reaction which included the expression of crisis, loss and disruption), and finally, (3) as transcendence (which represents the patient's embracement of their present situation, as well as being open to a different and altered future). The dichotomy of hope can be seen in patient's expression of hopelessness (an aspect apparent in individuals with MND as identified above) or in their hope for a cure. Hopelessness may be easily experienced for individuals with MND, several reasons may contribute to this, for instance, the disease can be controlling and force passivity, in addition to this individuals can be vulnerable to isolation, frustration, and negative thoughts (Ozanne et al., 2011). Alternatively, hope for a cure can be found in individuals, including hope for a medical cure, from a wish, expectation or a miracle from an individual's faith in God (Fanos et al., 2008). The paradox of chronic illness illustrates the central importance of acceptance for individuals with neurological conditions (Soundy et al., 2013). Indeed, in patients with MND, acceptance is regarded as the most common coping strategy used by patients (Hogg et al., 1994; Montel et al., 2012). Alternatively, examples of transcendence have been identified in patients with MND, for instance, patients with MND are able to positively reframe their situation (Montel et al., 2012). Further to this, evidence that can integrate patients experiences of MND would be extremely useful to the current literature base and could have wider application to other chronic or palliative illnesses. Thus, the purpose of the current review is to consider and synthesize common experiences of MND and better understand the effects of the illness on the patient's mental well-being and generalized hope.

## Methods

A thematic synthesis (Thomas and Harden, 2008) was chosen for this review. This method often includes a larger number of studies compared to other qualitative review approaches. One advantage of this is that it helps develop the thematic structure at the level of minor themes or codes (Soundy et al., 2014d). For the purposes of this work we position ourselves as subtle realists (Pope et al., 2007), one reason for this is because we seek to consider the common experiences, perceptions or expressions of individuals. However, we recognize that the synthesis only represents “a truth” and not the truth about the experiences of MND (Weed, 2008).

### Search strategy

The primary author conducted a systematic search of electronic databases from inception until June 2014. The following databases were utilized: CINAHL, AMED, Medline, Embase, PsychINFO, and Sports Discus. The key words utilized included; qualitative OR mixed methods AND experience OR expectation OR Hope AND Motor Neuron Disease Or Amyotrophic Lateral Sclerosis. Further searches included citation chasing of included articles.

#### Eligibility criteria

Articles were included when they satisfied the following eligibility criteria, considered within the domains of the “SPIDER” search tool (Cooke et al., 2012).

#### S—sample

Studies were included that focused on patients with MND.

#### PI—phenomenon of interest

Articles were included when they considered the patient's experience of having MND or other people's (spouse, family, or health care professionals) perception of the patient's experience. Articles were required to focus on and report the domains which related to mental well-being or generalized hopes.

#### D—design

Any type of qualitative design was considered including phenomenology, grounded theory, or ethnography. Mixed methods articles were included where a clear qualitative section was presented. Articles were excluded if they were case studies or reflective pieces; the volume of literature available meant saturation of themes would be achieved without such articles being included. Quantitative research, reviews, books, theses, and conference proceedings were excluded. Internet sources were excluded if not presented in a traditional article form with a methods section which could be critically evaluated.

#### E—evaluation

Articles were required to include interviews or focus groups and document experiences, views, or attitudes from users or other people (see above). Where articles reported both patient and carer experiences, articles were only included if data that related to the patient could be separately identified.

#### R—result type

To be included, articles had to contain qualitative results. Articles had to be published in English.

### Consolidated criteria for reporting qualitative studies (COREQ)

The COREQ (Tong et al., 2007) was selected to highlight the quality of reporting in past studies as well as to identify any studies that were considered “fatally flawed.” A fatal flaw is defined as a methodological weakness that compromises the trustworthiness of the data (Dixon-Woods et al., 2007). The COREQ contains three domains: The first domain has 8 items which focus on the research team and reflexivity, identifying the positionality of the team, as well as, how and if they are introduced and known to participants. The second domain has 15 items, addressing the methodological orientation of the paper and key processes typically used in methods. The third domain has 9 items which consider the process and procedures of analysis. An adapted form was used following a blind analysis of three random included studies by both authors. A kappa score was calculated (kappa = 0.43, *T* = 4.3, *p* < 0.001) and differences in responses compared. Items were adapted where required to clarify scoring (1 point for an item being present, 0 points where it is unclear or absent). The finalized form is available from first author.

### Synthesis

Initially open coding was used in an inductive approach to analysis without a-priori concepts intentionally being included. The coding would sometimes use *in vivo* coding to help retain meaning. At the same time, “idea webbing” (Arai et al., 2007; Popay et al., 2007) was used to question how concepts, themes, and sub-themes link together. The sub-themes and codes were then mapped into a framework of themes based on previous findings, relating to the model of hope enablement (Soundy et al., 2014d), generalized hopes (Soundy et al., 2014a) and factors which influence hope (Soundy et al., 2014c). A model was generated using an “idea web” (Arai et al., 2007; Popay et al., 2007) and “concept mapping” (Pope et al., 2007). The model in essence fuses the thematic structure together, making it simplified and focused.

## Results

To aid the flow and readability of the synthesis Section Synthesis articles are summarized by numbers^1^

### Descriptive

Twenty nine articles (Cobb and Hamera, 1986; Cox, 1992; Young and McNicoll, 1998; Brown, 2003; Murphy, 2004; Hughes et al., 2005; Brott et al., 2006; Hugel et al., 2006; Vitale and Genge, 2006; Brown and Addington-Hall, 2007; Foley et al., 2007, 2014a,b; Fanos et al., 2008; King et al., 2009; Locock et al., 2009, 2012; Locock and Brown, 2010; Gysels and Higginson, 2011; O'Brien et al., 2011, 2012; Ozanne et al., 2011; Hogden et al., 2012; Mckelvey et al., 2012; Whitehead et al., 2012; Gibbons et al., 2013; Mistry and Simpson, 2013; Pavey et al., 2013; Allen-Collinson and Pavey, 2014) were identified following the search. This included a total of 342 patients diagnosed with MND (175 male, 117 female, 50 unknown). Six studies used previously collected data. Mean ages reported 12 studies were between 42 and 68 years and age ranges in studies (reported in *n* = 17 studies) were between 30 and 80 years. The average time since diagnosis (when reported, *n* = 6 studies) ranged between 5 and 40 months. The most frequent location for interview (when reported, *n* = 18 studies) was reported in the participants home. The most frequent geographical location for studies were in the UK (*n* = 14), followed by the USA (*n* = 3), Australia (*n* = 2), and Canada (*n* = 2). Two studies identified cognitive impairment as exclusion criteria (Cox, 1992; Hughes et al., 2005). Two studies explicitly stated that cognitive impairment was not assessed (Murphy, 2004; Brown and Addington-Hall, 2007), although in one study (Brown and Addington-Hall, 2007) patients claimed no cognitive impairment. Other studies identified criteria that may have limited patients who had cognitive impairment. For instance, other criteria included patients who were; willing and able to communicate (Gysels and Higginson, 2011; King et al., 2009), have speech that was understandable (Ozanne et al., 2011), have English language conversational skills (Brott et al., 2006), or be able to “participate” (Allen-Collinson and Pavey, 2014). No additional reference within studies was given to the possible influence of cognitive impairment on results. Supplementary File A contains a figure representing the PRISMA flow diagram and Supplementary File B contains a summary table of included studies.

### Between study analyses using COREQ

The average score for the COREQ assessment of studies was 17/32 (*SD* ± 4). Within domain 1 only one study (1/29) identified what the researcher's occupation was at the time of the interview or what training and experience the interviewer had received. Only four studies (4/29) identified if a relationship was established prior to conducting the study and only two studies (2/29) identified what the interviewer characteristics were. Within domain 2 considering study design only seven (7/29) used field notes and only six studies (6/29) considered data saturation. Within domain three considering analysis and findings only two studies (2/29) considered details of a coding tree or audit trail and only four studies (4/29) identified minor themes. Ten studies (10/29) scored less than 16 and were discussed for exclusion; however no study was identified as fatally flawed. The complete COREQ assessment table is available from the first author.

### Synthesis

Five main themes were identified including; (1) The effects of the disease on interactions, relationships, roles and meaningful activities, (2) Responses that relate to the expression of hope, (3) Factors which disable hope, (4) Factors which enable hope, and (5) Cognitive and Practical adaption that enabled hope, control and coping. In order to obtain the most salient themes (in line with our positionality) codes were reported when at least three studies identified them.

#### Theme 1: the effects of the disease on interactions, relationships, roles, and meaningful activities

Individuals expressed specific and significant losses across a range of sub-themes identified below.

#### Loss and change to personal, social, and occupational relationships

Significant changes for many patients included functional losses that impacted on their vocational, occupational and family roles (1, 7, 9, 11, 16, 18, 20). This could include several roles, for instance, undertaking DIY, being the wage earner for the family, undertaking cooking, or walking children to school. The family role of father, mother, grandparent typically changed by assuming aspects of a patient-carer relationship. An important further change reported was the priority that patients placed on meaningful relationships, typically within the family and spending time with close others (1, 7, 9, 20, 29). Second, the most significant reference to loss included losses to what individuals called “normality” which was detailed as meaningful occupations, voluntary positions, hobbies, travel, pastimes and leisure activities (1, 5, 7, 8, 9, 11, 12, 16, 18, 20, 21, 29). Losses were identified by studies as relating to loss in an individual's social identity or sense of self (7, 11, 13, 16, 17, 18, 19, 25, 26). For instance, one study identified the loss from illness as an “abruption” or a sudden ending of one's previous life or as a “death sentence” (18).

#### Physical and functional losses and the future implication of loss

The losses to social roles, activities and interactions were related to the reporting of physical and functional loss (1, 2, 3, 7, 8, 9, 10, 11, 12, 16, 17, 18, 20, 26, 28), as well as future worries and what the loss would mean for others (1, 2, 6, 9, 10, 11, 12, 13, 15, 18, 25, 28). Description in articles illustrated the main losses and detail the moments when this was experienced by individuals. The losses include functioning of the legs, arms, communication, breathing, and fatigue, and would often be placed in context by individuals for instance once study identified that: “*Some participants worried about the practicalities of getting to the toilet, or managing drinking or eating, in an unfamiliar environment*” (17). The loss of future contact and relationships were expressed including; not seeing other family members as they grew up without the patient as a support figure, the loss that related to dependency and need for others to undertake even basic tasks for the patient and the ability to cope with such changes.

#### Theme 2: responses relating to the expression of hope

Several expressions of hope were identified by patients. The sub-themes group these responses together.

#### Disorientated response

First, individuals expressed shock, confusion and disbelief at the experiences of symptoms, as well as at diagnosis (2, 13, 14, 15, 17, 19, 20, 21, 23, 26). Symptoms were often and initially identified by individuals as relating to another diagnosis or dismissed for the seriousness of them. However, even when individuals had a better idea of the diagnosis there was still a sense of disbelief and the situation being surreal as well as being devastating. The psychological reactions that appeared to exist over a longer term included the inability to control emotions (11, 15, 16, 18, 23, 25, 26), this included; laughing at feeling helpless, not being able to stop crying, or fluctuating between happiness and sorrow. Patients would also express going between great optimism and despair (2, 18, 26) and ask the question “why me?,” not being able to comprehend or have an answer to the question (15, 16, 21, 26).

#### Responses that disabled hope and coping

Individuals associated the diagnosis with a loss of hope and the hopelessness of living (7, 13, 14, 15, 16, 17, 19, 27). Hope was also affected by increasing loss; however the incurable nature of the disease could create this perception. The hopelessness of their situation extended to the individuals hopes for their life (11, 16, 18, 19, 21, 25) including; the things they had wanted to do or achieve, the hope of possible change, the hope of being able to fight against the illness. Individuals specifically identified a perception of “powerlessness” or the inability to do anything about their situation (2, 11, 13, 14, 15, 19). For some, the disease had ruined the ability to live life (1, 9, 16, 20, 23), indeed patients could identify or report a point in life where they would be better off dead, or at which they would want to die, because at such a stage the suffering was deemed unacceptable (1, 2, 6, 7, 16, 18, 28). This may represent a hope for the suffering to end.

#### Defiance or challenging the illness

Individuals were able to defy and challenge the illness by positively adapting to it (see Theme 4 and 5). Defiance could be expressed however as a form of denial (2, 3, 5, 14, 15, 17, 21, 23, 26). For instance, patients may not be able to accept the diagnosis given and this could be related to the sense of shock of having the diagnosis, disbelief that the situation is real, not feeling that anything was wrong, not wanting to know, or not wanting to associate with any others with the illness. Individuals also wanted to defy the illness progression with the aim of curing themselves or being able to survive for longer (2, 5, 11).

#### Acknowledgement and acceptance

An initial stage of acceptance revolved around patients acknowledging the problem without resigning their life to it or embracing it (2, 5, 6, 7, 9, 12, 13, 18, 19, 23, 25). It represented an awareness of the reality of the disease, but also represented some psychological distance from it. This type of acceptance may have been used as a way to avoid thinking about the implications of the illness, or, not thinking too far into the future. The largest consideration to acceptance could be described as a resignation to acceptance (2, 5, 6, 7, 8, 9, 10, 11, 12, 13, 18, 19, 21, 23, 26, 29), akin to chronic sorrow, this type of acceptance was informed by the uncontrollable nature of the disease and the certain outcome which they faced. This reflected the patient's perception that they were powerless to the outcome and control the disease had in their live.

#### Embracement or transcendence

Another response closely linked to acceptance that related to resignation but was not identified in such a negative manner was the need to embrace it (2, 6, 9, 10, 11, 13, 15, 16, 18, 19, 20, 21, 23, 25, 29). This expression was associated with focusing on what could be done to aid their situation, including seeing life now as being as good as it can be.

#### Particularized hopes

Several different forms of particularized hopes were identified including; beating the odds, or that a miracle could occur in reality, or, a wish that 1 day they would wake up and be cured that some abnormality in their condition could assist this (4, 11, 17, 19, 23, 26, 27). A number of patients hoped in a cure from medicine and hoped that advancements in treatments would aid this (2, 4, 16, 21, 26, 27). Finally, some hoped in slowing progression of the illness or for a plateau in progression (16, 19, 25, 27, 28).

#### Theme 3: factors which disable hope

Patients identified several factors that acted to disable and negatively influence their hopes.

#### Interactions isolation and relationships

The disease had a significant impact on the actions, interactions and relatedness to others. Patients identified an awareness of meta-perceptions (5, 6, 11, 12, 15, 17, 20, 21, 25). This was typically a negative perception of how others viewed them or an aspect relating to them. Patients appeared more likely to distance themselves from social encounters as a result; there were embarrassment or negative feelings over one's speech and communication style for instance fear of being seen as being “drunk” rather than with an illness, being seen in a wheelchair, or the reliance on others in particular situations. Experiences of stigma were reported and often linked to speech, falling in public and other categories related to the effects of the illness (9, 11, 12, 15, 20, 21, 25). Patients could identify a downwards social comparison/relatedness with peers (4, 14, 16, 17). The reason for this type of comparison was that patients could experience distress at seeing their peers at a more advanced stage of the illness and understanding that this could be them in the future. Others didn't like the content of what was discussed, the nature, or intended purpose of such groups, often preferring to do more “normal” group activities which were not associated with MND. Further to this, some studies identified the negative impact on the patient of watching close others experience their deterioration (13, 15, 18).

Interactions that lacked respect and dignity (1, 11, 12, 13, 14, 19, 23, 25, 26, 28) were perceived by individuals with MND. Such interactions were identified as lacking emotional support and care. For instance, whilst in hospital patients reported feeling that their only use was that of someone that is useful for teaching purposes of medical students. In a similar way, other patients reported interactions that made them feel like a number rather than a person, with medical interactions focusing on the disease presentation. A lack of emotional support and care could leave patients feeling isolated and vulnerable. Several reason for this was attributed to the lack of empathy from staff, staff not giving time to talk about treatment options or advice, a misunderstanding or no understanding of the condition.

A significant lack of satisfaction with interactions and delivery of information was reported by patients (5, 7, 10, 15, 16, 20, 21, 23, 24, 25, 26, 28). One such example was patient's experiences of the referral and interaction process; including uncertainty to the service entitlement, limited time to digest the information or privacy. Other problems highlighted included feeling over-whelmed by information or support once the diagnosis had been given. Finally, individuals identified problems with the continuity of care (13, 14, 15, 19, 20, 24, 28), this included interactions that occurred or was absent between different health care teams and services. Other problems were identified as needing to fight to receive services and there being little consistency in the care team that visited or support individuals.

#### Problems with informational support

A number of studies identified a general lack of understanding of what MND was (3, 10, 12, 15, 16, 23), most often this was related to health care professionals, but also related to the general public. This could result in patients not wanting to approach health care professionals or not wanting to feel like they had to explain themselves to others who didn't understand. Other problems experienced during interactions with health care professionals included failure to diagnose, giving the wrong diagnosis, or not being willing to disclose diagnosis within the interaction. Further to this, being able to give the patient information relating to prognosis, including practical solutions on how to deal with aspects of the disease, utilizing technology that could assist the individual, advances in treatments and access services. As a result of this, information from health care professionals could be considered as questionable (3, 4, 6, 10, 13, 14, 16, 21, 22, 23, 26, 28).

#### Loss of control, agency, and autonomy

The process of losing control to the disease as it progresses was significant for patients and was identified as a factor which could only get worse (2, 3, 6, 7, 11, 12, 13, 19, 21). The loss of control could limit the choices of activities individuals participated in and acted to prevented interactions. The uncertainty of the progressive decline and not perceiving what losses were approaching them (pre and post diagnosis), as well as the length of life that is left (post diagnosis) was consistently identified. The fear of death and how a patient would die could be expressed as a severe worry for instance, having difficulties swallowing, or an inability to express oneself when dying. Thus, the effect on, and importance of autonomy was consistently identified as a factor across different stages of the illness which influenced the patient's mental well-being (1, 2, 7, 10, 12, 13, 15, 16, 21).

As the illness progressed, the patients' life became more dependent on others, this meant a life that was more repetitive, limited physically, functionally and socially, and of less interest because of the limitations in autonomy. At later stages of the disease the dependence on others was extremely challenging and impacting on patients (2, 5, 7, 12, 13, 15, 18, 21, 25, 28). The extent of losses in independence meant carers were required to perform basic tasks, making patients feel like a burden and vulnerable. The reason for this was identified as a loss in their privacy and patients would often feel guilty about the stress placed on others. Related to this, patients identified that the disease limited interaction and support through its progressive and intrusive nature, such as not being able to communicate (1, 10, 12, 13, 15, 16, 17, 18, 20, 21, 22, 25, 29). For instance, less individuals who visited the patient, the patient or/and spouse having to give up work, going places or visiting people was made more difficult and could be solely dependent on the support of others, interaction was interrupted, and being part of a conversation was difficult and became less reciprocal in the benefits perceived, or less satisfying and could cease. Further to this, friends could change their interaction with the patient and challenges with spouses could create strain, sadness or even martial break down at the loss of communication.

Finally, individuals experience problems accepting and using functional or technological devices (1, 2, 6, 7, 9, 11, 12, 15, 16, 19, 21, 22). These problems included accepting what the device would mean to them and facing the losses they had experienced or giving up control to the illness, similarly the device could affect an individual self-esteem or image. Further, frustration was caused when the device didn't appear to work effectively.

#### Experience of negative emotions

Patients could feel and express a range of negative emotions. This included being frustrated (2, 8, 11, 12, 15, 21, 25) at the effects of the illness. This was created by different factors, for instance, taking a long time to get ready, having deteriorated speech, inability to engage in activities once enjoyed and other losses which were important. Patients could also feel sadness and grief about the losses the illness had imposed on their lives (1, 5, 12).

#### Theme 4: factors which enable hope

Patients identified several factors which were identified as aiding, supporting or enabling hope.

#### The importance of autonomy, control, and agency

Patients highly valued maximizing the autonomy of their actions and task functions and required that they were respected within interactions (5, 7, 8, 11, 12, 14, 15, 16, 18, 19, 20, 21, 23, 24, 25, 28). Frustration was generated from carers, health care professionals and others undertaking tasks for the patients, assuming they wanted help to undertake the task, or undertake the task at a quicker rate. Also health care professionals that introduced assistive aids e.g., wheelchairs without asking, or medical decisions like having a gastrostomy feed fitted with consulting the patient were deemed as lacking respect and removing autonomy from the patient. The value of autonomy appeared to be that it allowed patients to feel in control, where possible to allow patients to feel a sense of “normalcy” in being independent and stop others imposing and restricting their lifestyle. Losing the capacity to talk was a particular worry which would limit autonomy and control significantly. Related to this, patients consistently highlighted the need for choice and autonomy given during an interaction (5, 6, 7, 9, 12, 14, 15, 20, 24, 25, 26, 28). Within medical care this meant interactions needed to be worded in a way that offered the patient a choice of decision. Bad experiences combined with the need for perceived control could alter the way the patient engaged with services. For instance, patients could exert their control by deciding when to engage with services. Other forms of control may be challenged by others and members of the family telling the patient what they can or can't do before they are ready to lose that role or task, for instance, cooking in the kitchen, getting dressed. Individuals could feel fear and anxiety about their future deterioration, as well as being afraid of death (2, 10, 11, 12, 13, 14, 15, 21, 23, 25, 26, 27, 28). Finally, having control over the end of life decisions was also identified (7, 12, 14, 20, 27, 28). This included euthanasia, decisions about resuscitation or assisted suicide, and circumstances of death like dying at home and having the presence of certain individuals like specialist nurses.

#### Determination, agency, and useful emotions

Patients identified the need to fight against the illness and not give into it (7, 11, 19). Others identified the need for patient endurance of their situation and having resilience against it, combined with a determination to continue to live (2, 4, 7, 13, 14).

#### Valued social interactions and support

Patients could relate to peers who were in a similar situation (2, 16, 17, 19, 25, 29). Such individuals could be seen as a role model, and were considered important because they were “similar” others (mostly but not necessarily individuals with MND) who were able to understand their situation. Further to this and more generally, relationships and interaction with others was identified as important (3, 5, 11, 14, 16, 18, 19, 20, 25, 27, 29). This was primarily identified as the care and emotional support received from close others (typically individuals within the family unit), this could include being there for the patient at difficult times and sharing and valuing them. In some cases for spouses having to care for a patient meant being closer and intimate with them and growing closer together. Examples of this can be seen in two spouses (20); one stated “*Leon still told me that he loved me. He'd write me little love emails*.” The other stated “*Kevin would always show me that he loved with his touching*.”

The continued expression of strong emotions or feelings, like anger, love through closeness and sexual intimacy was identified as important (1, 7, 13, 16, 20). Emotional support from friends and from health care professionals was identified too, although, not so consistently. The priority of maintaining and using the time left for relationships and interactions with close others was something which became evermore important for individuals (1, 4, 5, 7, 14, 15, 16, 18, 19, 20, 23, 25, 29). The family could enable coping and become a reason to live and continue, or to use medical interventions. In a similar way, peers friends and close others became important to individuals (4, 5, 8, 12, 16, 20, 29). Apart from the benefits of being connected and valued with others, interactions were also beneficial in that they provided a chance for individuals to give back or care for others (5, 16, 17, 18, 19, 27). This allowed individuals to see a sense of purpose from the greater good. Examples of this included making an awareness film about MND, being supportive of others, helping others (like children) accept the patient's death or praying for others.

Patients described the need to retain dignity and feel respected during interactions (5, 7, 12, 13, 15, 23, 25). For instance, having interactions in a private place and seeing that interactions are valued and heard was important. One lady stated “*he (GP) didn't believe me (about symptoms) and he just sort of sent me away and said…* ‘*stop drinking your wine*”’ (23). Having health care professionals that were sensitive to the losses were important, for instance, understanding that simple tasks like using a commode can be degrading. A further characteristic valued by patients was the “nature” of other's highlighting the value of individuals who were personable, reassuring and who remained calm (4, 6, 10). Other studies documented the value of humor for patients (4, 11, 16, 18, 20, 29), this included black humor or teasing. For instance, when a friend, who hadn't visited in a while, came around the patient said “*Hello, where the hell have you been?*” (20).

The role and care given by HCPs was identified as important in that support from health care professionals influenced participants mental well-being and coping (4, 5, 12, 14, 16, 23, 26). Health care professionals, including, and notably, professionals from specialized charities were identified as important in providing emotional support as well as decision making. Importantly, patients cited the importance of reassurance and trust from health care professionals (6, 9, 10, 14, 23, 28). Trust was gained by experiencing professionals who were caring and sensitive to the patients' needs. This was particularly important for end of life care (6, 11, 14, 28).

#### Use and value of tangible support

A number of studies highlighted that patients required physical assistance in increasing quantities (5, 11, 16, 18). This resulted in patients identifying a need for support services and access and utilization of functional or technological aids. A number of patients highlighted the need for different support services which provided tangible assistance to care (3, 11, 14, 16, 17, 21, 25, 26). This included personal care assistance/health care attendance at home, charity services, or care from the hospital. The cost of care could restrict care provision (3, 10, 11, 20). Functional aids were valued by patients (3, 7, 9, 11, 12, 16, 17, 18, 20, 21, 22), this most frequently included systems which aid communication such as an alternative and augmentative communication device. It also included assistive devices that maintained function (e.g., hoists), or devices that enabled continuation of activities (e.g., wheelchairs or mobility scooter), as well as home computers that enabled interaction across the internet.

#### Theme 5: cognitive and practical adaptation that enabled hope, control, and coping

Patients identified specific strategies which were used to help them undertake a more self-controlled and dominant response to their losses.

#### Cognitive adaptation

Several strategies were used by patients in dealing with the symptoms and problems they faced, including: (a) Bringing time closer and focusing on the ability to cope with the present circumstances (2, 10, 14, 18, 25, 27). This meant focusing on coping day by day or week by week, because the presence was more preferable to consider than the future. (b) Dealing with problems as they arrive and affect their life (2, 7, 10, 11, 12, 14, 18, 25, 29). To some extent this limited focus on uncontrollable changes and losses. (c) Focusing on what could be done or achieved (2, 5, 6, 7, 11, 12, 13, 14, 27), rather than what they couldn't or what they had lost. This was seen as enhancing the access of what could be considered “normal.” (d) Planning activities and interactions to engage in (4, 8, 11, 12, 14, 20, 27). (e) Using religious faith to aid acceptance, and coping, or hoping for a miracle to occur, or life after death (5, 9, 14, 16, 20, 27, 29). (f) Having the responsibility choice and ownership of how to view the illness and situation. This meant realizing that there was a choice of how to view their situation and that there was a need to make the most of their present circumstances (2, 5, 7, 10, 11, 14, 16, 18, 20, 27, 29),

#### Pragmatic adaptation

Patients often suggested that the best they could do was to live positively and make the most of the time they had left (2, 10, 11, 14, 15, 18, 20, 27, 29). For instance, doing activities or visiting places that they had wanted to go to but never had. Other individuals wanted a distraction or a break from the illness (7, 10, 11, 14, 17). This was achieved by pretending it is not there, to avoid negative thoughts about what it meant in their life, not reading literature available or associating with MND groups. Other pragmatic adaptation included individuals continuing meaningful activities (4, 5, 7, 9, 10, 12, 14, 15, 16, 17, 18, 19, 20, 25, 27, 29) or continuing being “normal” (1, 2, 5, 7, 9, 17, 18, 24) which provided access to a sense of purpose and meaning as well as valued interactions.

#### Taking action and searching to enable control

In order to promote a perceived sense of control over the illness taking action and searching provided a sense of purpose and meaning. Individuals consistently identified the need to search for information in order to feel more in control (2, 4, 5, 9, 10, 12, 13, 14, 15, 22, 25, 26), but also take action as a way of controlling aspects of their life that were controllable (2, 4, 5, 11, 12, 14, 18, 19). This included, using alternative therapies (2, 11, 16), adapting health behaviors including rehabilitative exercises, considering care routines, or undertaking hobbies or activities (5, 11, 16) and making dietary adaption (2, 9, 16).

#### Application of findings to a model: adapting the model of hope enablement (MHE)

**Figure 1:**
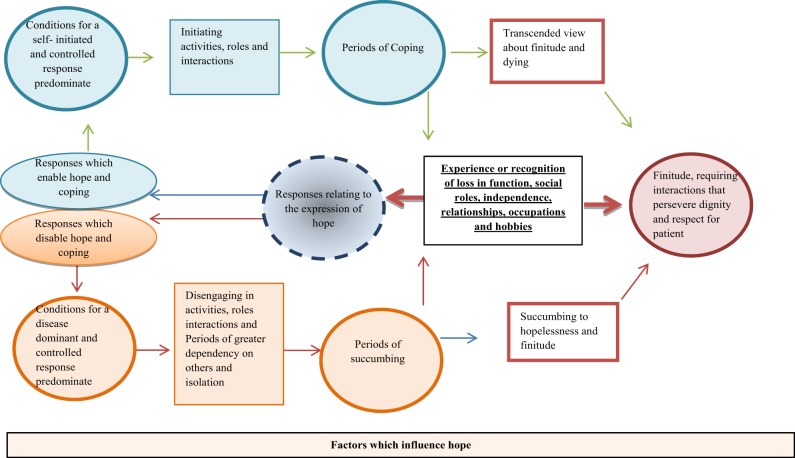
**The Adapted Hope Enablement Model (HEM)**.

**Figure 2:**
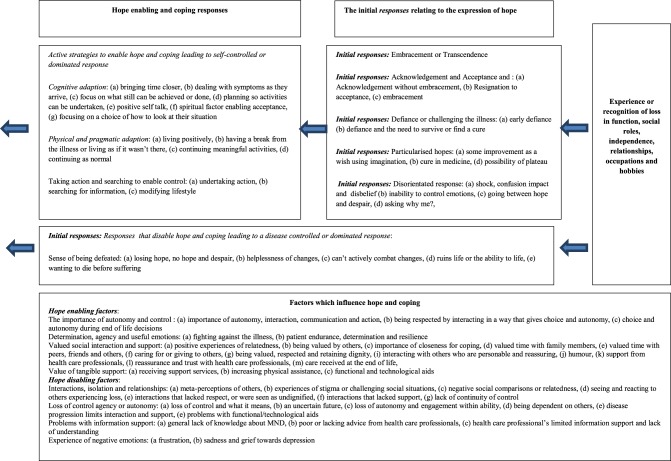
**Describing the processes that lead to a predominantly self-controlled/led or disease controlled/led response**.

The MHE (see Figures 1, 2) is focused on (re)establishing the losses experienced in a patient's life including function, roles, independence, relationships, occupations and hobbies. The model proposes two types of responses to loss which include: (a) a more self-dominant, self-controlled and has the individual as active in that response, alternatively, (b) a more disease/illness dominant and disease controlled response which leaves the individual more passive is possible. The model also identifies factors which influence this. Both responses and the factor which influence hope are detailed above and summarized below.

#### Factors which may influence the experience of hope

Several factors may influence how loss is experienced and the individual's expression of hope and ability to cope. The above synthesis and list of factors illustrate this. Central factors include the importance of being independent and autonomous for as long as possible, as well as the importance of social support and positive relationships with family and others. This is supported by previous research (Soundy et al., 2014c).

#### The expression of hope and a more self-initiated and controlled response

This response is made up of several components, exclusively or in conjunction with one another; (a) an acknowledgement, or acceptance of what has happened and understanding of the meaning within their life. Part of this likely requires an acceptance that they are not able to control their situation, as this in itself aids the manageability of the MND (Ozanne et al., 2011). (b) Some form of transcendence (Soundy et al., 2013), for instance, positive cognitive reappraisal or benefit finding of one's situation (Nowlan et al., 2014), or post traumatic growth (Tedeschi and Calhoun, 2004). In essence responses which enables the patient to want to keep living or, an understanding that life may be as good as it can be, or that the experience of the disease provides opportunity for meaning and purpose. (c) The possibility of hope for change, no change, or for less worsening or change of symptoms, useful because it is not a concrete hope and represents a preparedness of the patient to accept what is hoped for may not occur (Soundy et al., 2014b). (d) Some form of a denial related response and/or inability to accept the present circumstances including having a break from the illness, having a future concrete hope of a cure, or rejecting the illness situation. Within patients with MND, denial has been suggested to control the amount of reality that individuals are faced with at any one time; within the current model it clearly provides access to a period of coping. Although, it can be considered problematic if it is continuously used as a strategy (Centers, 2001),

The self-initiated responses are likely to be associated with a degree of agency (Snyder et al., 1991) including want to continue to fight, engage or appreciate living. Further, these responses will also likely be associated with a greater potential for individuals to initiate activities, valued roles and interactions (linked to occupational, voluntary, family, and social) and hence allow factors which enable mental well-being, hope and autonomy to be fostered. Importantly, this response leads to a set of cognitive, pragmatic and action orientated strategies which enable access to engage in meaningful interactions and relationships. In other words, such a response accompanied with specific strategies which allow and promote the following to occur: (1) a sense of meaning and purpose, (2) personal and loving connection, belonging, unity and role fulfillment (family, social, or occupational), (3) a sense of perceived control through more autonomous activity, (4) pleasure, positive feelings and emotions to be generated. For those who have obtained a more transcended view it may be this period of coping extends until nearer death.

#### The expression of hope and a more disease-dominant and controlled response

The experiences of loss may be perceived as too much, disorientating, overwhelming or past a point of deterioration that is acceptable. The disease controlled response is enforced by the control taken by the illness and the dependency required. Importantly research has recognized the perceived controllability of a stressor as a crucial dimension which influences a patient's coping response (Compas et al., 2012). The disease dominant response is likely informed by the expression of hope and would likely include: (a) A denial of the effects or inability to accept the disease and its meaning because of shock and an inability to comprehend the situation. (b) A consideration of the uncertainty about one's present and future situation and what it means, leading to worry and fear. (c) Resignation to certain future prospects caused by the illness, with a focus on the impossibility of it changing. Thus, what was considered once possibility is now considered impossible. (d) Acknowledging present or future loss beyond the ability or want to live. (e) Being overcome or disorientated by emotions such as fear and worry when considering the implications of the illness.

These responses are likely associated with, or, have a greater chance of producing disengagement in activities and interactions as well as producing greater periods of dependency on others and isolation. This allows or promotes the following to occur; (1) reduced access to meaningful activities which provide a sense of purpose, (2) a sense that they are in an uncontrollable, unpredictable situation or disease controlled situation, (3) further isolation as connection and unity with others may be challenged. Thus, before another loss is experienced individuals may experience a period of succumbing to the illness, less mental well-being as well as hopelessness about their situation. This may be an aspect which is hard to challenge thus the period of this experience may lead to or continue until a patient's death.

## Discussion

The current review has been able to bring together existing information on the experiences of MND, identifying what is known, not known and what further work is needed. Further to this, the research has been able to identify a model of hope enablement combining the expression of hope with cognitive strategies and factors which influence the (re)access of generalized hopes. Importantly, this research identifies that a period of coping is possible for patients and that social support, as well as retaining independence and autonomy are very important for patients with MND.

### Interactions, relationships and social support

The current review illustrates that care and loving interactions are especially important from family and close others for maintaining mental well-being. However, the current review supports previous literature which has identified the possibility and danger of a loss of intimacy in relationships as the disease progresses, due to role changes and dependency (Goldstein et al., 1998). Previous literature has identified that social support has a strong association with quality of life (Lulé et al., 2012), is inversely related to a wish to die (McLeod and Clarke, 2007), and has an increasing importance as the disease progresses (Neudert et al., 2001). Thus, there is real value in being able to maintain close and intimate communication with an individual's spouse and family members, because as the disease progresses these relationships, interactions and connections provide a source of hope and comfort for the patient, as well as a sense of meaning in life which likely influences the patient's mental well-being. They are so important because they can last until the patient's death providing a unique source of hope. However, past literature has suggested that patients with a younger age may find greater fluctuations in social support with disease progression (Ray and Street, 2005).

The role and value of peers in the current review highlighted that patients may benefit from groups. Several benefits have been noted by past literature for instance, peers can give advice on how to manage with the disability, or claim benefits or make adaptions to the home. Further to this, being able to help others can provide a sense of satisfaction and being able to relate to others can give a sense of camaraderie, finally seeing others in a worse state but managing could be inspiring (Locock and Brown, 2010). However, being able to access and benefit from the groups could be restricted by several factors including; restrictions imposed by the disease, the location of the group (requiring travel which can't be made), or that patients may not want to identify or socialize with the group. Further, patients may be negatively affected by the loss of peers after a relationship has been established.

Support services can be limited by insufficient homecare, limited access to different members of the multi-disciplinary team, lack of knowledge about MND, diagnostic delays, as well as delivery of the diagnosis (Foley et al., 2012). The consequences of this has been identified in previous literature, for instance late referral to palliative services may negatively impact on the patients quality of life (Bede et al., 2011). In a similar way the lack of awareness of specific care services can have a negative impact on the patient, for instance, within end of life care, health care professionals have identified a lack of awareness of the Preferred Priorities for Care document (Preston et al., 2011). Finally, again in line with the current findings, patients with MND can be dissatisfied with the tangible and informational support provided by health care professionals (Foley et al., 2012). However, strategies to change this have been identified in the current review.

### Considerations for hope

The continued experience of loss and the rapid decline in individuals physical and functional health may explain why individuals can feel hopeless and experience a loss of meaning in life as psychological consequences of MND (Blackhall, 2012). If hope was associated purely with an individual's survival then the experience of hopelessness would be the natural outcome, however, literature considering patients with MND suggests this is not the case. For instance, Centers (2001) describes a hope accessed by patients which is more meaningful than a hope for survival which includes “*a peaceful acceptance of life, and its inexplicable beginnings and endings*” (p. 260), she also notes that hope is tied to the ability to find meaning, this includes connection and loving relationships with others or with God. However, patients in the current review could identify a point where loss was too much or the suffering to great and thus hope for an end to suffering, which has previously been identified as the highest level of hope.

### Considerations around strategies that enabled hope

Living with MND often means patients are continually required to adjust to new losses (McLeod and Clarke, 2007). Much of the losses experienced by patients with MND required them frequently interpret and reinterpret the meaning of that loss in their life. Past the expression of hope, individuals broadly identified strategies that were used to enable management of their condition on a daily basis. These strategies may be used to deal with losses which fundamentally challenged their hopefulness, it may be the first or initial stages that promote mental well-being for patients. Much of these strategies could be considered within secondary control or accommodative coping which includes adapting to stress by reappraisal, acceptance, distraction and positive thinking and, to a lesser extent, passive or disengagement coping which includes ways to avoid stress (Compas et al., 2012). There is evidence of the value of such processes, such as focusing on life in the present moment rather than the future (Centers, 2001). Alternatively, having a break from the reality of their situation may help patients manage their emotions for instance by engaging in activities like playing video games (Roger et al., 2014). Perhaps the most important strategy is positive reappraisal, for instance, in older adults, positive reappraisal can be positively associated with improvements in positive emotions, social relationships, depression and life satisfaction (Nowlan et al., 2014). Further to this, recent literature (Hu et al., 2014) has identified a significant association between mental health (similar to mental well-being defined above) and positive reappraisal. Having a break or avoidance of the illness such as playing video games, listening to music or watching TV has been reported to allow patients to change the meaning of their situation when the reality cannot be changed.

The final set of hope enabling or coping strategies included taking action and searching for meaning and purpose, searching for information and understanding, controlling the controllable aspects of their life, undertaking activities, hobbies or past times, and continuing meaningful activities, some of which could be “normal.” To some extent this has been identified previously, for instance, positive effects have been identified in individuals who engage in activities that are social (rather than vocational) such as groups, clubs or classes (McCabe and O'Connor, 2012), although access to work in individuals with brain disorders has also been identified as beneficial (Hartley et al., 2014), the stage and deterioration of patients with MND may not make this accessible. Alternatively, benefit has been found in those who take action by searching for information and gaining understanding (Abdulla et al., 2014). Thus being able to access a more self-initiated response to the illness creates access to meaning and purpose in life and generates mental well-being. It should be noted that many of the strategies identified may have followed some kind of acknowledgement or acceptance from the patient about their situation. Thus, it can be said that acknowledgement and acceptance may lead on and allow patients with MND to more positively approach there situation. This has been found to be an important characteristic in individuals with neurological conditions who are considered to adjust better to their situation (McCabe and O'Connor, 2012).

### Considerations to the model of hope enablement

The recent MHE identified by Soundy et al. (2014d) has been substantially updated to include and consider how the expression of hope (Soundy et al., 2013), factors which influence hope (Soundy et al., 2014c) and strategies used by individuals to access generalized hopes (Soundy et al., 2014a) interact and impact on the original model proposed. The current model suggests that periods of coping are possible for patients with MND and that these periods will likely improve a patient's mental well-being. Further, it suggests that these periods are obtained through (re)accessing the sources of generalized hope for individuals. It has also identified the importance for patients of retaining a sense of control over their life situation and circumstances.

### Implications for rehabilitation

Health care professionals and patients alike will be able to consider the model and process that accompany the model. This will aid them to consider how they can engage with patients in a way that allows the patients dignity, respect and promotes autonomy. It is important to note that the current review has not accounted for the impact of cognitive impairment and it is worth noting that a spectrum of cognitive changes will affect patients psychological processes, although only few individuals experience dementia (Achi and Rudnicki, 2012). For instance, the prevalence of frontotemporal dementia has been identified in around 8.1% (CI: 5.6–11.5%) of patients, with apathy, disinhibition and perseveration being the most commonly reported changes (Raaphorst et al., 2012). Thus, where cognitive impairments (Lillo and Hodges, 2009) allow, patients with MND may be able to consider the value of different cognitive strategies from the results and understanding the different way other individuals (re)access there generalized hopes.

### Limitations

The analysis and model may have been limited by the first author's knowledge and perception or view of what was contained within the data selected. Unique findings may have been lost because of the type of synthesis used and the positionality taken and the use of a framework to guide the later stages of analysis. That said, the data itself may not reflect the less common experiences of individuals with MND or be able to detail change over time. The critical appraisal of studies identified poor reporting practices, with confidence in individual studies limited by a lack of minor themes and reporting of data saturation. It was not possible to quantify the level of cognitive impairment of the included patients and thus it wasn't possible to determine if and how cognitive impairment would influence individual's hope and mental well-being. It is possible that cognitive impairment could negatively impact on individual's relationships, interactions and meaningful activities and disable hope. Cognitive impairment may also impact individual's cognitive adaptation and emotional expression.

### Conflict of interest statement

The authors declare that the research was conducted in the absence of any commercial or financial relationships that could be construed as a potential conflict of interest.

## Footnotes

^1^Note the following numbers are used to represent articles within Section Synthesis: 1, Allen-Collinson and Pavey, 2014; 2, Brown and Addington-Hall, 2007; 3, Cox, 1992; 4, Fanos et al., 2008; 5, Foley et al., 2007; 6, Foley et al., 2014a; 7, Foley et al., 2014b; 8, Gibbons et al., 2013; 9, Gysels and Higginson, 2011; 10, Hughes et al., 2005; 11, King et al., 2009; 12, Brott et al., 2006;, 13, Brown, 2003; 14, Hogden et al., 2012; 15, Hugel et al., 2006; 16, Cobb and Hamera, 1986; 17, Locock and Brown, 2010; 18, Locock et al., 2009; 19, Locock et al., 2012; 20, Mckelvey et al., 2012; 21, Mistry and Simpson, 2013; 22, Murphy, 2004; 23, O'Brien et al., 2011; 24, O'Brien et al., 2012;, 25, Ozanne et al., 2011; 26, Pavey et al., 2013; 27, Vitale and Genge, 2006; 28, Whitehead et al., 2012; 29, Young and McNicoll, 1998.
