# Supplementary material for: Patients experiences of maintaining mental well-being and hope within motor neuron disease: a thematic synthesis
Source: Front Psychol. 2015 May 12;6:606. doi: 10.3389/fpsyg.2015.00606 (PMC4428059; doi:10.3389/fpsyg.2015.00606)
Supplement: Supplementary file 1 [file Table1.DOCX]

Supplementary File B

Table 1 The study characteristics of included studies

| **Study** | **Qualitative method** | **Participants** | **Sampling and setting** | **Data collection, study aims and analysis** |
| --- | --- | --- | --- | --- |
| Allen-Collinson and Pavey (2013) | Semi-structured interviews | 42 (31♂) patients with MND  Mean age: not given  Age range: 40 to 74 years (16 undisclosed)  Mean time with illness: not given  Mean time until diagnosis: not given | Sampling: purposive  Eligibility: (a) formal clinical diagnosis of MND at > 6 months prior to agreeing to participate; and (b) able to participate in a face-to-face interview or via an e-mail interview.  Setting: primarily in patients homes, but place identified by patients. “sometimes” accompanied by partner or family member.  Study location: UK | Data collection tool: 11 question interview guide    Study Aim**:** “*undertake an in-depth sociological study*  *of the lived experience of MND amongst patients who had been clinically diagnosed with MND*” (page 6)  Average interview time: not given  Interview time range: not given  Analysis: phenomenological analysis |
| Brown and Addington-Hall (2007) | Narrative Case studies | 13 (9♂) patients with MND  8 with ALS  2 with progressive bulbar palsy  1 with progressive muscular atrophy  2 with primary lateral sclerosis  Mean age: not given  Age range: 39 to 85 years  Mean time with illness: not given  Mean time until diagnosis: not given | Sampling: not stated.  Eligibility: (a) ‘firm’ diagnosis of MND, (b) could communicate at point of recruitment, (c) over 18 years of age.  Setting: patient’s own home or care home  Study location: UK | Data collection tool: interview constructed in three parts: (1) living and coping (2) further detail about particular issues (3) generic questions.  Study Aim**: “***The aim of the study was to explore patient experiences and how they talk about living and coping with MND*.” (page 202)  Average interview time: not given  Interview time range: not given  Analysis: narrative |
| Cox (1992) | Semi-structured interviews | 10 (?♂) patients with MND  10 carers  8 occupational therapists  Mean age: 63 years  Age range: 39 to 74 years  Mean time with illness: not given  Mean time until diagnosis: not given | Sampling: not stated.  Eligibility: not stated.  Setting: homes of the participants.  Study location: UK | Data collection tool: not given  Study Aim**:** “examine the perceived everyday needs as viewed by the persons with MND, their carer and their OT. The aim was to explore the day-to-day needs and requirements of the person with MND” (page 334)  Average interview time: not given  Interview time range: not given  Analysis: not stated. Possibly content analysis. |
| Fanos et al (2008) | Semi-structured interviews | 16 (13♂) patients with MND  Mean age: 54 years  Age range: 36 to 72 years  Mean time with illness: 38 months from diagnosis.  Mean time until diagnosis: 14 months | Sampling: convenience  Eligibility: (a) families at a one centre  Setting: clinic during a visit.  Study location: USA | Data collection tool: individuals were asked to describe the meaning of hope for them.  Study Aim**: “***explore hope in patients with ALS*” (page 471)  Average interview time: not given  Interview time range: not given  Analysis: content analysis. |
| Foley et al (2007) | ‘Open ended questions’ used in interviews | 5 (?♂) patients with ALS  Mean age: not given  Age range: not given  Mean time with illness: not given  Mean time until diagnosis: not given | Sampling: purposive  Eligibility: (a) selected by health care professionals  Setting: participants home.  Study location: UK | Data collection tool: open ended questions developed around quality of life  Study Aim**:** “*first, to explore self-referent ‘meaning’ of QoL as perceived by individuals with ALS patients, and secondly, to explore how their experience of health care affected their subjective well-being*” (page 164)  Average interview time: not given  Interview time range: not given  Analysis: Colaizz’s 7-step method (thematic analysis) |
| Foley et al (2014) | Grounded theory | 34 (17♂) patients with MND  Mean age: not given  Age range: 20 to 81 years  Mean time with illness: 31 months  Mean time until diagnosis: not given | Sampling: purposive and theoretical.  Eligibility: (a) on a previous MND research register.  Setting: 32 at home. 1 in hospice and 1 in nursing home  Study location: UK | Data collection tool: consider experiences from the ‘ground up’ guide not pre-prepared.  Study Aim**: “***identify from a MND population based*  *cohort, key psycho-social processes that underpin*  *how people with MND engage with their services*” (p 319)  Average interview time: 1 hour and 10 mins  Average time range: 40 mins to 2 hours and 10 mins  Analysis: open, axial and selective coding as with grounded theory. |
| Foley et al (2014b)  **same details as above* | Grounded theory | 34 (17♂) patients with MND  Mean age: not given  Age range: 20 to 81 years  Mean time with illness: 31 months  Mean time until diagnosis: not given | Sampling: purposive and theoretical.  Eligibility: (a) on a previous MND research register.  Setting: 32 at home. 1 in hospice and 1 in nursing home  Study location: UK | Data collection tool: consider experiences from the ‘ground up’ guide not pre-prepared.  Study Aim**: “***further our understanding of what loss means to people with ALS and how people with ALS exert control in their care in response to loss*.” (page 114)  Average interview time: 1 hour and 10 mins  Average time range: 40 mins to 2 hours and 10 mins  Analysis: open, axial and selective coding as with grounded theory. |
| Gibbons et al (2013) | Interpretative phenomenological analysis | 10 (7♂) patients with MND  Mean age: 60 years  Age range:  Mean time with illness: not given  Mean time until diagnosis: not given | Sampling: purposive  Eligibility: (a) ‘fatigued’ participants that expressed fatigue as an issue were approached.  Setting: participants home  Study location: UK | Data collection tool: interview schedule focused around functional limitations, cognitive effects, fatigue development, social effects, negative affect, learned response and relieving factors.  Study Aim**: “***provide both a phenomenological account of fatigue in motor neurone disease and offer a definition that may be used as the basis for future qualitative work to develop a disease-specific questionnaire for fatigue*.” (page 2)  Average interview time: not given  Interview time range: 15 to 45 minutes  Analysis: Interpretative phenomenological analysis |
| Gyseles and Higginson (2011) | In-depth and semi-structured interviews | 10 (9♂) patients with MND  Mean age: not given. Median 42  Age range: 24 to 77 years  Mean time with illness: not given  Mean time until diagnosis: not given | Sampling: purposive  Eligibility: (a) currently experiencing problems of breathlessness, priority on those with palliative care needs.  Setting: participants home, except 2 who were interviewed at the researchers office (n=2)  Study location: UK | Data collection tool: explored the experience of breathlessness and care towards this symptoms from others.  Study Aim**: “***We aimed to explore and compare the lived experience of breathlessness for patients with four conditions -COPD, heart failure, cancer and MND- all with heavy symptom burden, poor prognoses, high breathlessness rates and palliative care needs*.” (page 2)  Average interview time: 65 minutes  Interview time range: 40 to 150 minutes  Analysis: Concept of explanatory models as a framework for analysis |
| Hughes et al (2005) | semi-structured interviews | 9 (6♂) patients with MND  Mean age: not given  Age range: 30 to 70+ years  Mean time with illness: not given. 4 greater than 4 years or less than 1 year and 1 between 2-3 years..  Mean time until diagnosis: not given | Sampling: purposive and snowball  Eligibility: (a) no impaired mental status (b) not involved in other research (c) good command of English.  Setting: individuals homes except one individual  Study location: UK | Data collection tool: interview considered three broad topics; (1) experiences of services, (2) living with MND and its impact, (3) suggestions for service change.  Study Aim**: “***understand people’s experiences*  *and to generate ideas for developing practice and*  *policy in MND health, social and palliative care*” (page 65)  Average interview time: not given  Interview time range: 25 to 90 mins  Analysis: thematic |
| King et al (2009) | Grounded theory | 25 (17♂) patients with MND  Mean age: not given  Age range: not given.  Time with illness: not given.  Mean time until diagnosis: not given | Sampling: theoretical  Eligibility: (a) willing and able to communicate.  Setting: participants in their homes or 3 in nursing homes  Study location: Australia | Data collection tool: initial question what has been happening since you were diagnosed with MND is asked then followed up in aground up approach.  Study Aim**: “***understand what it is like to live with the illness and how people with the disease negotiate and make choices about their ongoing care*” (page 476)  Average interview time: not given  Interview time range: not given  Analysis: open, axial and selective coding and software. |
| Brott et al (2006) | In-depth interviews | 7 (3♂) patients with MND  Mean age: not given  Age range: not given  Mean time with illness: 8 months to 4 years  Mean time until diagnosis: not given | Sampling: convenience  Eligibility: (a) diagnosed with MND at least 6 months prior to taking part, (b) living in the community, (c) having English language skills.  Setting: participants home  Study location: New Zealand | Data collection tool: broad starting point of when participants realised something wasn’t quite right  Study Aim**:** “*What is the lived experience of people with motor neurone disease*?” (page 25)  Average interview time: not given  Interview time range: not given  Analysis: narrative |
| Brown (2003) | In-depth interviews | 6 (2♂) patients with MND  Mean age: 53 years  Age range: 38 to 69 years  Mean time with illness: not given  Mean time until diagnosis: not given | Sampling: purposive (not stated)  Eligibility: (a) confirmed diagnosis (b) willingness to participate  Setting: not disclosed  Study location: UK | Data collection tool: primary question of what it is like to have MND was then followed up with considerations to caring experiences.  Study Aim**: “***gain understanding of caring experiences in MND in order to contribute to further development of caring policy and practice*” (Page 208)  Average interview time: not given  Interview time range: 60 to 90 minutes  Analysis: Hermeneutic analysis |
| Hogden et al (2012) | Semi-structured interviews | 14 (7♂) patients with ALS  Mean age: 60.5 years  Age range: 40 to 77 years  Mean time with illness: 32 months. Range 2 – 93 months  Mean time until diagnosis: not given | Sampling: convenience  Eligibility: (a) had to attend one of two clinics. No further information given.  Setting: clinic  Study location: Australia | Data collection tool: focused on the experiences of specialised multi-disciplinary clinical ALS services.  Study Aim**: “***investigate decision-making from the perspective of patients within specialized multidisci­plinary clinical ALS services in an attempt to identify factors that influence decision-making for symptom management and quality of life*” (Page 830)  Average interview time: not given  Interview time range: not given  Analysis: QSR NVIVO 9 |
| Hugel et al (2006) | Interpretive phenomenological analysis | 13 (9♂) patients with MND  Mean age: 64 years  Age range: 33 to 79 years  Mean time with illness:  Mean time until diagnosis: 8 months (range 3 to 60 months) | Sampling: convenience  Eligibility: (a) newly diagnosis within 6 weeks of diagnosis.  Setting: neurosciences centre.  Study location: UK | Data collection tool: all interviews began by asking the patient how they have been since diagnosis  Study Aim**:** “*explore issues surrounding a new diagnosis for patients diagnosed with MND at a large regional neurosciences centre*” (page 161)  Average interview time: not given  Interview time range: not given  Analysis: Interpretative phenomenological analysis |
| Kuckelman-Cobb and Hamera (1986) | In-depth interviews | XX (XX♂) patients with MND  Mean age: not given  Age range:  Mean time with illness: not given  Mean time until diagnosis: not given | Sampling: purposive  Eligibility: (a) 3 signs of ALS (b) diagnosis within the past 6 months (c) individuals in the fifth and sixth decade of life (d) at least one male participant.  Setting: participants homes  Study location: USA | Data collection tool: focus on individuals ideas of causation of the illness, changes in relationships with family and friends, use of illness role models, utilisation and evaluation of various kinds of treatment, spiritual changes and symptoms experience.  Study Aim**: “***describe…the social context of ALS, then present two separate cases*” (page 643)  Average interview time: not given  Interview time range: not given  Analysis: case by case analysis |
| Locock and Brown (2010)  *reanalysis from Brown and Addingington-Hall (2007) and Locock et al (2009) | Not detailed. | 48 (?♂) patients with MND  Mean age: not given  Age range: not given.  Mean time with illness: not given  Mean time until diagnosis: not given | Sampling: based on other research.  Eligibility: not applicable as based on previous research  Setting: previous data analysed again.  Study location: UK | Data collection tool:  Study Aim**:**  Average interview time: not given  Interview time range: not given  Analysis: amplified analysis and suprea analysis. |
| Locock et al (2009) | Narrative interviews and semi-structured interviews | 35 (?♂) patients with MND  11 carer givers  Mean age: not given  Age range: not given  Mean time with illness: not given  Mean time until diagnosis: not given | Sampling: maximum variation  Eligibility: (a) people living with MND  Setting: participants home  Study location: UK | Data collection tool:  Study Aim**: “***examines the relevance of biographical disruption and repair to the experience of MND*” (page 1046)  Average interview time: not given  Interview time range: 1 to 2 hours  Analysis: thematic analysis |
| Locock et al (2012)  *reanalysis of above data. | Narrative interviews and semi-structured interviews | 35 (?♂) patients with MND  11 carer givers  Mean age: not given  Age range: not given  Mean time with illness: not given.  Mean time until diagnosis: not given | Sampling: maximum variation  Eligibility: (a) people living with MND  Setting: participants home  Study location: UK | Data collection tool:  Study Aim**: “***examines the relevance of biographical disruption and repair to the experience of MND*” (page 1046)  Average interview time: not given  Interview time range: 1 to 2 hours  Analysis: Secondary analysis |
| McKelvey et al (2012) | Semi-structured interviews | 7 (2♂) surviving spouses of patients with MND  Mean age: not given  Age range: 46 to 65 years.  Mean time with illness: 40 months until death (range 10 to 78 months)  Mean time until diagnosis: not given | Sampling: Purposive  Eligibility: (a) spouses of people with ALS, (b) native English speakers (c) had no known neurological deficits  Setting: not given.  Study location: USA | Data collection tool: 18 question protocol around experiences.  Study Aim**: “***describe communication patterns of individuals with ALS over time as the disease progressed, and to understand the lived human experiences from the surviving spouses’ perspectives, which were provided after their spouses’ deaths*” (page 233)  Average interview time: not given  Interview time range: not given  Analysis: thematic |
| Mistry and Simpson (2013) | Semi-structured interview | 7 (3♂) patients with MND  Mean age: 68 years  Age range: 58 to 75 years  Mean time with illness: 5 months  Mean time until diagnosis: not given | Sampling: convenience  Eligibility: (a) consultant confirmed diagnosis within past 6 months (b) aged over 18 years (c) not other neurological signs.  Setting: participants own home  Study location: UK | Data collection tool: questions around diagnosis process, subsequent hospital visit, and the participants behaviours, thoughts, emotions and attitude about the diagnosis up to the point of interview.  Study Aim**: “***explore the transitional process*  *and experience of participants as they moved from receiving a diagnosis through to exploring coping strategies*” (page 941)  Average interview time: not given  Interview time range: not given  Analysis: Interpretative phenomenological analysis |
| Murphy et al (2004) | Open conversations and semi-structured interviews | 15 (8♂) patients with MND  Mean age: 63 years  Age range: 45 to 78 years  Mean time with illness: not given  Mean time until diagnosis: not given | Sampling: random from register  Eligibility: (a) diagnosis of MND  Setting: participants own home  Study location: UK | Data collection tool: consider perception of communication with MND and consider the use of assisted communication devices  Study Aim**: “***One of the aims of the project was to examine the communication strategies of 15 people with MND (specifically)…the use of AAC, and the participants’ perceptions about AAC*.” (page 261)  Average interview time: not given  Interview time range: not given  Analysis: thematic analysis |
| O’Brien et al (2011) | Narrative interviews | 24 (9♂) patients with MND  28 carers  Mean age: 61 years  Age range: 25 to 84 years  Mean time with illness: 23 months  Mean time until diagnosis: not given | Sampling: maximum purposive  Eligibility: (a) diagnosis of ALS or MND  Setting: participants own home  Study location: UK | Data collection tool: in depth questions around the experiences leading up to and the following the diagnosis.  Study Aim**:** “*better understand the period from symptom onset to diagnosis from the perspective of those living with ALS/MND*” (Page 98)  Average interview time: not given  Interview time range: 45 minutes to 2 hours  Analysis: QSR NVIVO |
| O’Brien et al (2011b)  *same data as O’Brian et al (2011) | Mixed methods audit | 24 (9♂) patients with MND  Mean age: 61 years  Age range: 25 to 84 years  Mean time with illness: 23 months  Mean time until diagnosis: not given | Sampling: purposive  Eligibility: (a) review of case notes within one location of individuals with MND  Setting: N/A – case notes  Study location: UK | Data collection tool: in depth questions around the experiences leading up to and the following the diagnosis.  Study Aim**: “***A greater understanding of the barriers to service uptake may provide a basis for increasing homecare provision with consequent benefits for patients, carers and the NHS*.” (Page 124)  Average interview time: not given  Interview time range: 45 minutes to 2 hours  Analysis: thematic |
| Ozanne et al (2011) | Semi-structured interviews | 14 (7♂) patients with MND  Mean age: not given. Median 67.5 years.  Age range: 42 to 80 years  Mean time with illness: not given  Mean time until diagnosis: not given | Sampling: maximum variation  Eligibility: (a) diagnosed 6 months prior to entering the study.(b) speech understandable or be able to provide written communication to allow full communication (c) not at a terminal stage (d) no respiratory insufficiency  Setting: participants home or at hosptial  Study location: Sweden | Data collection tool: Questions focused around theeffect of disease, ability to manage  Study Aim**: “***illuminate factors that facilitate and hinder the manageability of living with ALS for both patients and next of kin*.” (page 1365)  Average interview time: median 48 minutes  Interview time range: 20-83 minutes  Analysis: content analysis |
| Pavey et al (2011)  *data from Allen-Collinson and Pavey (2013) | Semi-structured interviews | 42 (31♂) patients with MND 16 undisclosed  Mean age: not given  Age range: 42 to 74 years  Mean time with illness: not given  Mean time until diagnosis: not given | Eligibility: (a) formal clinical diagnosis of MND at > 6 months prior to agreeing to participate; and (b) able to participate in a face-to-face interview or via an e-mail interview.  Setting: primarily in patients homes, but place identified by patients. “sometimes” accompanied by partner or family member.  Study location: UK | Data collection tool: considered (a) life before diagnosis (b) receiving diagnosis (c)life after diagnosis  Study Aim**: “***undertake a sociological-phenomenological study of the lived experience of MND*” (page 2)  Average interview time: not given  Interview time range: not given  Analysis: thematic analysis |
| Vitale and Genge (2006) | Mixed methods | 13 (9♂) patients with MND  Mean age: 55 years  Age range: 35 to 73 years  Mean time with illness: 11 months (3 to 22 months)  Mean time until diagnosis: not given | Sampling: purposive  Eligibility: (a) excluded clinician diagnosis of patients with depression (b) above 18 years (c) speak English or French  Setting: clinic  Study location: Canada | Data collection tool: focused around hope and what it means to individuals and how it changes over time. Probing used to explore response.  Study Aim**: “**step towards understanding the experience of hope in ALS” (page 28)  Average interview time: not given  Interview time range: 45 to 60 minutes  Analysis: thematic |
| Whitehead et al (2011)  *same data as O’Brian et al (2011) | Semi-structured | 24 (9♂) patients with MND  28 carers  Mean age: 61 years  Age range: 25 to 84 years  Mean time with illness: 23 months  Mean time until diagnosis: not given | Sampling: maximum purposive  Eligibility: (a) diagnosis of ALS or MND  Setting: participants own home  Study location: UK | Data collection tool: in depth questions around the experiences leading up to and the following the diagnosis.  Study Aim**: “***explore the experiences of people with MND, as well as current and bereaved family carers, of the final stages of the disease*” (page 369)  Average interview time: not given  Interview time range: 45 minutes to 2 hours  Analysis: thematic analysis |
| Young and McNicoll (1998) | Semi-structured interviews | 13 (7♂) patients with ALS  Mean age: not given  Age range: 36 to 81 years  Mean time with illness: 1 to 24 years  Mean time until diagnosis: not given | Sampling: purposive  Eligibility: (a) patients with “advanced” ALS who were making an exceptionally positive adaptation  Setting: participants homes  Study location: Canada | Data collection tool: 5 questions, quality of life before the illness, current positive life experiences, personal and social resources and the emotional impact of the interview.  Study Aim**:** “*highlighting the strengths and positive aspects in the lives of people with ALS*” (page 36)  Average interview time: not given  Interview time range: not given  Analysis: constant comparative approach |

*Note: MND = Motor Neurone Disease, ALS = Amyotrophic Lateral Sclerosis, Mins = minutes
