# Supplementary figures and images for: Patients experiences of maintaining mental well-being and hope within motor neuron disease: a thematic synthesis
Source: Front Psychol. 2015 May 12;6:606. doi: 10.3389/fpsyg.2015.00606 (PMC4428059; doi:10.3389/fpsyg.2015.00606)

Figure 1 A PRISMA diagram for the study

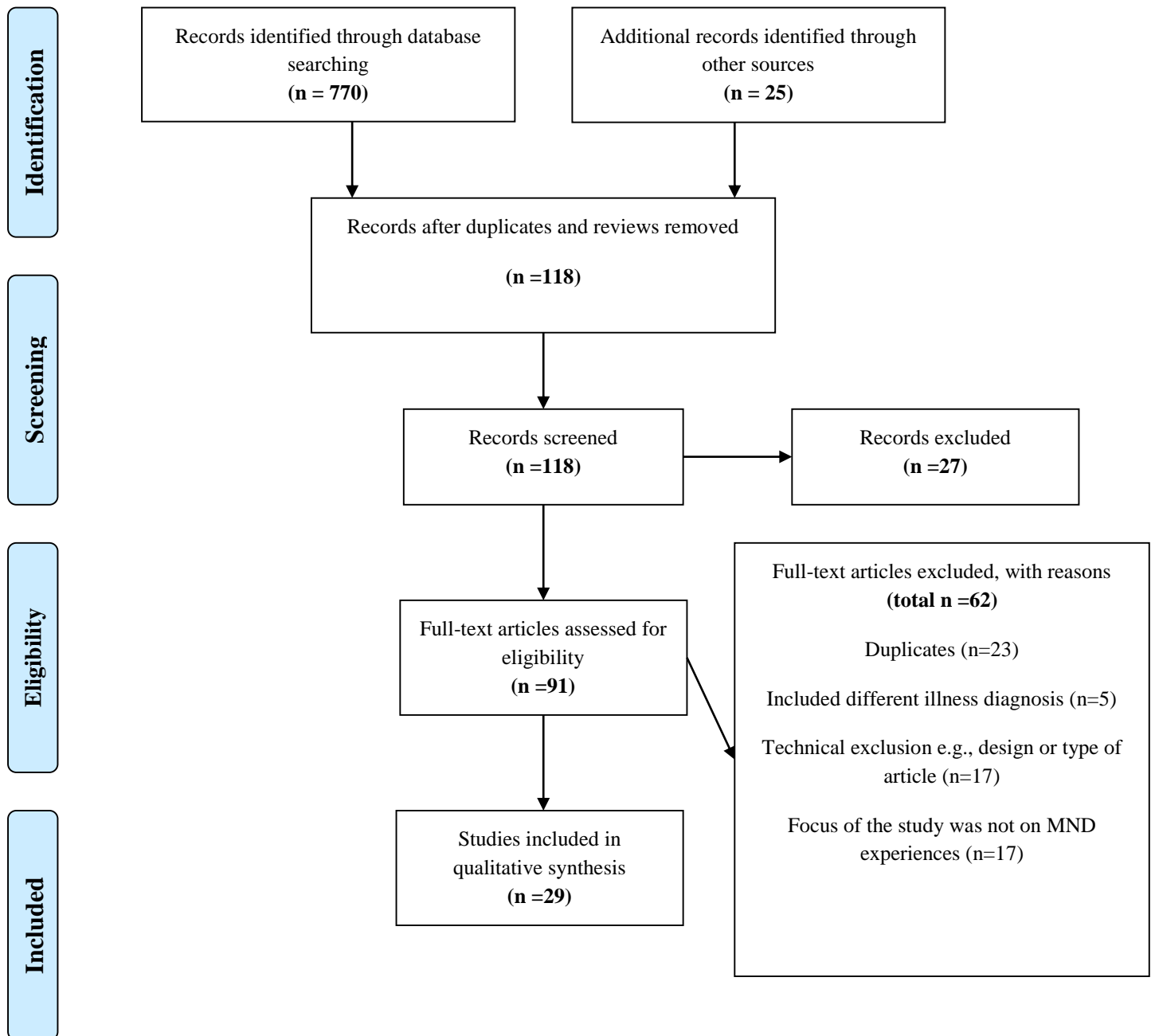

Supplement: Supplementary file 2 [file Image1.PDF]
